# Contrasting effects of peroxisome-proliferator-activated receptor (PPAR)γ agonists on membrane-associated prostaglandin E_2 _synthase-1 in IL-1β-stimulated rat chondrocytes: evidence for PPARγ-independent inhibition by 15-deoxy-Δ^12,14^prostaglandin J_2_

**DOI:** 10.1186/ar1830

**Published:** 2005-09-22

**Authors:** Arnaud Bianchi, David Moulin, Sylvie Sebillaud, Meriem Koufany, Marie-Madeleine Galteau, Patrick Netter, Bernard Terlain, Jean-Yves Jouzeau

**Affiliations:** 1 Laboratoire de Physiopathologie et Pharmacologie Articulaires, UMR 7561 CNRS-UHP, 54505 Vandœuvre-lès-Nancy, France

## Abstract

Microsomal prostaglandin E synthase (mPGES)-1 is a newly identified inducible enzyme of the arachidonic acid cascade with a key function in prostaglandin (PG)E_2 _synthesis. We investigated the kinetics of inducible cyclo-oxygenase (COX)-2 and mPGES-1 expression with respect to the production of 6-keto-PGF_1α _and PGE_2 _in rat chondrocytes stimulated with 10 ng/ml IL-1β, and compared their modulation by peroxisome-proliferator-activated receptor (PPAR)γ agonists. Real-time PCR analysis showed that IL-1β induced COX-2 expression maximally (37-fold) at 12 hours and mPGES-1 expression maximally (68-fold) at 24 hours. Levels of 6-keto-PGF_1α _and PGE_2 _peaked 24 hours after stimulation with IL-1β; the induction of PGE_2 _was greater (11-fold versus 70-fold, respectively). The cyclopentenone 15-deoxy-Δ^12,14^prostaglandin J_2 _(15d-PGJ_2_) decreased prostaglandin synthesis in a dose-dependent manner (0.1 to 10 μM), with more potency on PGE_2 _level than on 6-keto-PGF_1α _level (-90% versus -66% at 10 μM). A high dose of 15d-PGJ_2 _partly decreased COX-2 expression but decreased mPGES-1 expression almost completely at both the mRNA and protein levels. Rosiglitazone was poorly effective on these parameters even at 10 μM. Inhibitory effects of 10 μM 15d-PGJ_2 _were neither reduced by PPARγ blockade with GW-9662 nor enhanced by PPARγ overexpression, supporting a PPARγ-independent mechanism. EMSA and TransAM^® ^analyses demonstrated that mutated IκBα almost completely suppressed the stimulating effect of IL-1β on mPGES-1 expression and PGE_2 _production, whereas 15d-PGJ_2 _inhibited NF-κB transactivation. These data demonstrate the following in IL-1-stimulated rat chondrocytes: first, mPGES-1 is rate limiting for PGE_2 _synthesis; second, activation of the prostaglandin cascade requires NF-κB activation; third, 15d-PGJ_2 _strongly inhibits the synthesis of prostaglandins, in contrast with rosiglitazone; fourth, inhibition by 15d-PGJ_2 _occurs independently of PPARγ through inhibition of the NF-κB pathway; fifth, mPGES-1 is the main target of 15d-PGJ_2_.

## Introduction

Prostaglandins (PGs) are well-known lipid mediators that reproduce the cardinal signs of inflammation [1] but also contribute to tumorigenesis, gastrointestinal protection or osteogenesis [2-5]. Decreasing their biosynthesis by the inhibition of cyclo-oxygenases (COXs) is thought to account for most of the therapeutical properties of non-steroidal anti-inflammatory drugs. During inflammation, the pathophysiological contribution of prostaglandins is supported by PGE_2_, the major mediator produced by monocytes in response to inflammatory stimulus, and prostacyclin (PGI_2_). However, since the discovery of at least two COX isoenzymes, the pathophysiological relevance of PG must be considered from a different point of view. First, inflammation can be ascribed to inducible COX-2-derived PG rather than to basal COX-1-derived PG [6]. Second, PGE_2 _and PGI_2 _are now recognized as end-point products of a coordinate enzymatic cascade comprising phospholipases A_2_, cyclooxygenases and terminal PG synthases whose activities are coupled preferentially between constitutive and inducible isoforms [7]. Third, PG produced by COX-2 switches from PGE_2 _to 15-deoxy-Δ^12,14^prostaglandin J_2 _(15d-PGJ_2_) in the course of acute inflammation [8]. Because 15d-PGJ_2_, a cyclopentenone by-product of PGD_2_, has shown anti-inflammatory properties in various experimental models [9,10], it has been proposed as an endogenous regulator of inflammation favouring the resolution of acute flares [11].

PGE synthase-1 (PGES-1), the enzyme converting the COX-derived PGH_2 _into PGE_2_, exists in multiple forms with distinct enzymatic properties, modes of expression, subcellular localizations and intracellular functions [12]. One of its isoforms, cPGES-1, is a cytosolic protein found as a complex with heat shock protein 90 (Hsp90) that is constitutively expressed in a wide variety of cells and tissues. Another isoform, microsomal PGE synthase-1 (mPGES-1), is a perinuclear membrane-associated protein belonging to the microsomal glutathione S-transferase family. In contrast with cPGES-1, its expression is induced by pro-inflammatory cytokines, growth factors, bacterial endotoxins and phorbol esters and is downregulated by anti-inflammatory corticosteroids [12]. As mentioned above, PGES-1 isoforms display distinct functional coupling with upstream COX in cells; cPGES-1 is predominantly coupled with constitutive COX-1, thereby contributing to basal PG synthesis, whereas mPGES-1 is preferentially linked with inducible COX-2 and contributes to stimulated PG synthesis [7]. Recently a novel PGES, mPGES-2 [13], was cloned and was shown to be highly expressed in heart and brain. Its role remains largely unknown, especially in inflammatory conditions.

Peroxisome-proliferator-activated receptor γ (PPARγ) is a ligand-activated nuclear transcription factor belonging to the nuclear hormone receptor superfamily. PPARγ binds, as a heterodimer with retinoid X receptor, to peroxisome-proliferator-response element (PPRE) located in the promoter of numerous target genes whose expression is regulated by PPARγ agonists. Agonists of PPARγ include synthetic ligands, as antidiabetic thiazolidinediones, and natural compounds, as fatty acids and 15d-PGJ_2_, which were shown initially to have a major function in adipocyte differentiation and glucose homeostasis [14-16]. However, PPARγ agonists were recently thought to contribute to the control of inflammation by inhibiting the transcriptional induction of pro-inflammatory cytokines (tumour necrosis factor-α, IL-1 and IL-6) or genes encoding inflammatory enzymes (inducible nitric oxide synthase and COX-2) in activated monocytic cells [17,18]. Similar pharmacological potencies were reported in chondrocytes [19] and synoviocytes [20] exposed to an inflammatory stimulus, giving a rationale to the anti-inflammatory effect of PPARγ agonists in experimental arthritis [10,21]. Because 15d-PGJ_2 _was thought to be a negative regulator of experimental inflammation [11], it is tempting to speculate that part of this effect could be supported by the regulation of PPARγ target genes, possibly through the control of transcription factors such as NF-κB or activator protein-1 [22,23].

Chondrocytes express both COX isoenzymes [24] and produce large amounts of eicosanoids under inflammatory conditions [25]. However, COX-2 represents only the first inducible step in the stimulated synthesis of PG [12] and its inhibition by PPARγ ligands remains moderate in articular cells [19,20]. We therefore investigated whether PPARγ agonists could reduce PG synthesis by inhibiting mPGES-1 in rat chondrocytes stimulated with IL-1β. Such a mechanism would be consistent with the ability of 15d-PGJ_2 _to inhibit PGE_2 _production and to downregulate mPGES-1 in microsomal fractions from CHO cells overexpressing mPGES [26].

The present study demonstrates an early induction of COX-2 and a delayed induction of mPGES-1 by IL-1β in rat chondrocytes, with the stimulated synthesis of prostaglandins fitting well the expression profile of mPGES-1 for PGE_2 _while remaining lower than the extent of COX-2 induction for 6-keto-PGF_1α _(the stable metabolite of PGI_2_). In our experimental system, 15d-PGJ_2 _lowered the 6-keto-PGF_1α _level and the expression of COX-2 but was much more potent towards the PGE_2 _level and the expression of mPGES-1, supporting the view that mPGES-1 is the rate-limiting step in PGE_2 _synthesis. The dose-dependent inhibitory potency of 15d-PGJ_2 _was not reproduced by the high-affinity PPARγ agonist rosiglitazone and was affected neither by blockade of PPARγ with the antagonist GW-9662 nor by PPARγ overexpression. Consistent with a PPARγ-independent mechanism was our final observation that 15d-PGJ_2 _decreased NF-κB transactivation, which is crucial for the induction of mPGES-1 and the stimulation of PGE_2 _synthesis by IL-1β in rat chondrocytes.

## Materials and methods

### Isolation and culture of rat chondrocytes

Chondrocytes were isolated from femoral heads of healthy Wistar male rats (130 to 150 g) (Charles River, Saint-Aubin-les-Elbeuf, France), killed under general anaesthesia (AErrane™; Baxter SA, Maurepas, France) in accordance with national animal care guidelines, after approval by our internal ethics committee. Cells were obtained by sequential digestion with pronase and collagenase [27], then washed twice in PBS and cultured to confluence in 75 cm^2 ^flasks at 37°C in a humidified atmosphere containing 5% CO_2_. The medium used was DMEM/Ham's F-12 supplemented with L-glutamine (2 mM), penicillin (100 U/ml), streptomycin (100 μg/ml) and either 10% heat-inactivated FCS (Life Technologies) during subculturing or 1% FCS during experiments. Chondrocytes were used between passages 1 and 3 to prevent dedifferentiation.

### Study design

Chondrocytes maintained in low (1%) FCS medium were stimulated with 10 ng/ml IL-1β (Sigma, St-Quentin-Fallavier, France) in the presence or absence (vehicle alone, 0.1% of final concentration in dimethylsulphoxide) of PPAR agonists added 4 hours before IL-1β. In a preliminary kinetic study, mRNA levels of COX-2 and mPGES-1 in cell layers were determined from 6 to 48 hours after challenge with IL-1β, whereas 6-keto-PGF_1α _and PGE_2 _levels were assayed from 6 to 36 hours in culture supernatants. Thereafter, COX-2 mRNA level was checked 12 hours after exposure to IL-1β, whereas the mPGES-1 mRNA level, the COX-2 and mPGES-1 protein levels, and the secreted 6-keto-PGF_1α _and PGE_2 _levels were evaluated at 24 hours. The PPARγ agonists rosiglitazone (Cayman, Ann Arbor, MI, USA) or 15d-PGJ_2 _(Calbiochem, Meudon, France) were used in the range 0.1 to 10 μM, whereas additional PPARγ agonist troglitazone (Cayman) and PPARγ antagonist GW-9662 (Cayman) were used at 10 μM.

### Assay for chondrocyte viability

Cell viability was assessed by the mitochondrial-dependent reduction of 3-(4,5-dimethylthiazol-2-yl)-2,5-diphenyl-2*H*-tetrazolium bromide (MTT; Sigma) into formazan [28]. In brief, cells were incubated for 24 hours at 37°C in the presence or absence of IL-1β and/or PPARγ agonists (added 4 hours before IL-1β) in low-FCS (1%) DMEM/Ham's F-12 medium. Chondrocytes were incubated further with MTT (1 mg/ml final concentration) for 4 hours at 37°C before the addition of lysing buffer (20% w/v SDS in a 50% aqueous solution of dimethylformamide, pH 4.7). After 24 hours of incubation at 37°C, solubilization of formazan crystals was quantified by measuring *A*_580 _on a Multiskan^® ^microplate reader (Labsystems, Montigny-le-Bretonneux, France).

### RNA extraction and real-time PCR analysis

Total RNA was isolated from chondrocyte layers using Trizol^® ^(Invitrogen, Cergy-Pontoise, France). Two micrograms of total RNA were reverse-transcribed for 90 minutes at 37°C with 200 U of Moloney Murine Leukaemia Virus reverse transcriptase (Invitrogen) and hexamer random primers. Expression of COX-2, mPGES-1 and adiponectin (chosen as a specific PPARγ target gene [29]) mRNAs were quantified by real-time PCR with the Lightcycler^® ^(Roche) technology and the SYBRgreen master mix system^® ^(Qiagen, Courtabœuf, France). After amplification, a melting curve was constructed to determine the melting temperature of each PCR product; their sizes were checked on a 2% agarose gel stained with ethidium bromide (0.5 μg/ml). Each run included standard dilutions and positive and negative reaction controls. The mRNA levels of each gene of interest and of the ribosomal protein S29, chosen as a housekeeping gene, were determined in parallel for each sample. Results are expressed as the normalized ratio of mRNA level of each gene of interest over the S29 gene.

The gene-specific primer pairs used were as follows: mPGES-1, sense 5'-TCGCCTGGATACATTTCCTC-3', antisense 5'-GTCCCCCATTGTGGTATCTG-3'; COX-2, sense 5'-TACAAGCAGTGGCAAAGGCC-3', antisense 5'-CAGTATTGAGGAGAACAGATGGG-3'; adiponectin, sense 5'-AATCCTGCCCAGTCATGAAG-3', antisense 5'-TCTCCAGGAGTGCCATCTCT-3'; S29, sense 5'-AAGATGGGTCACCAGCAGCTCTACG-3', antisense 5'-AGACGCGGCAAGAGCGAGAA-3'.

### Transient transfection experiments

Chondrocytes were seeded in six-well plates at 5 × 10^5 ^cells per well and grown to 80% confluence. Cells were transfected with either 500 ng of a PPARγ expression vector (pcDNA3.1 PPARγ, a gift from Dr H. Fahmi, Centre Hospitalier de l'Université de Montréal, Montréal, Canada), or 500 ng of a dominant-negative vector of NF-κB (IκBαΔN (Ala32, Ala36) from Clontech). Transfections were performed for 2 hours with 10 μl of polyethyleneimine reagent (Euromedex, Souffelweyersheim, France) in 1 ml of culture medium. At 24 hours after transfection, cells were stimulated with IL-1β for 24 hours in the presence or absence of PPARγ agonists.

### Preparation of nuclear extracts and electrophoretic mobility-shift assay (EMSA)

Nuclear proteins were isolated as described elsewhere [30] with minor modifications. In brief, cells were scraped in a lysis buffer (10 mM HEPES, pH 7.9, 10 mM KCl, 1 mM dithiothreitol (DTT)) containing a protease-inhibitor cocktail and 0.5% Igepal^®^, then incubated for 15 min on ice. Nuclei were collected by centrifugation at 2,000 *g *for 5 min at 4°C and resuspended in 50 μl of HEPES buffer without Igepal^® ^and KCl, but containing 420 mM NaCl. After a 30 min incubation on ice, nuclear debris were removed by centrifugation at 13,000 *g *for 10 min at 4°C; supernatants were collected and then stored at -80°C before use. The DNA sequences of the double-stranded oligonucleotides specific for NF-κB were 5'-GATCCAGTTGAGGGGACTTTCCCAGGCG-3' and 5'-GATCCGCCTGGGAAAGTCCCCTCAACTG-3'. Complementary strands were annealed and double-stranded oligonucleotides were labelled with [^32^P]dCTP by using the Klenow fragment of DNA polymerase (Invitrogen). Nuclear proteins (5 μg) were incubated for 10 min at 4°C in a binding buffer (20 mM Tris/HCl, pH 7.9, 5 mM MgCl_2_, 0.5 mM DTT, 0.5 mM EDTA and 20% glycerol) in the presence of 2 μg of poly(dIdC). The extracts were then incubated for 30 min at 4°C with 10,000 c.p.m. of ^32^P-labelled NF-κB probe. The samples were loaded on a 5% native polyacrylamide gel and run in 0.5 × Tris/borate/EDTA buffer. NF-κB-specific bands were confirmed by competition with a 100-fold excess of unlabelled probe, which resulted in no shifted band.

### NF-κB transactivation analysis

Nuclear proteins were prepared with the TransAM^® ^nuclear extract kit in accordance with the manufacturer's protocol (Active Motif Europe, Rixensart, Belgium). In brief, cells were scraped into PBS containing phosphatase and protease inhibitors, centrifuged, resuspended in 1 × hypotonic buffer and then kept on ice for 15 min. After the addition of detergent, lysates were centrifuged at 14,000 *g *for 30 s at 4°C. The pellets were resuspended in complete lysis buffer (20 mM HEPES, pH 7.5, 350 mM NaCl, 20% glycerol, 1% Igepal^®^, 1 mM MgCl_2_, 0.5 mM EDTA, 0.1 mM EGTA, 1 mM DTT, phosphatase and protease inhibitors) and shaken vigorously. After incubation on ice and centrifugation at 14,000 *g *for 10 min at 4°C, supernatants were collected and protein concentration was determined with a Bradford-based assay (Bio-Rad Laboratories, Marnes-la-Coquette, France).

NF-κB activation was determined with the TransAM^® ^ELISA kit (Active Motif Europe). In brief, 5 μg of nuclear extract was added to each well of a 96-well plate into which an oligonucleotide with a NF-κB consensus binding site had been immobilized. After 1 hour of incubation with smooth agitation, wells were washed three times with washing buffer (100 mM PBS, pH 7.5, 500 mM NaCl and 1% Tween 20) and then incubated with p65 antibody (dilution 1:1,000 in washing buffer) for 1 hour at 20°C. After three successive washings with buffer, the wells were finally incubated for 1 hour with diluted horseradish peroxidase-conjugated antibody (dilution 1:1,000 in washing buffer) before the addition of 100 μl of developing solution (3,3',5,5'-tetramethylbenzidine substrate solution diluted in 1% dimethylsulphoxide). After 5 min of incubation, the reaction was stopped by the addition of 100 μl of 0.5 M H_2_SO_4 _and the final *A*_450 _was read on a Multiskan^® ^microplate reader.

### Assays for PGE_2 _and 6-keto-PGF_1α_

Levels of PGE_2 _and 6-keto-PGF_1α _were determined in culture supernatants with Assay Design^® ^ELISA kits (Oxford Biomedical Research, Ann Arbor, MI, USA) in accordance with manufacturer's instructions. Assays are based on the combined use of a monoclonal antibody against PGE_2 _or PGF_1α _and an alkaline phosphatase-conjugated polyclonal antibody. After the addition of *p*-nitrophenyl phosphate substrate, *A*_405 _was read at on a micro Multiskan^® ^plate reader. The limits of detection were 10 pg/ml and 1.4 pg/ml for PGE_2 _and 6-keto-PGF_1α_, respectively, with negligible cross-reactivity with PGE_1 _and PGF_2α_, respectively (manufacturer's data). Positive controls were used in each experiment.

### Western blot analysis

Cells, seeded in six-well plates and grown to 90% confluence, were washed twice with ice-cold PBS and scraped off the wells in 1 × Laemmli blue for PPARγ or in TBS containing 0.1% SDS for other proteins. Cells were disrupted by sonication (five pulses) and centrifuged at 800 *g *for 10 min, before determination of protein concentration with a Bradford-based assay. Protein samples (5 μg) were analysed by SDS-PAGE (10% acrylamide for COX-2 and PPARγ, 12% for β-actin, and 15% for mPGES-1), and electroblotted on a poly(vinylidene difluoride) membrane. After 1 hour in blocking buffer (TBS-Tween with 5% nonfat dried milk), membranes (Immobilon; Waters, Saint-Quentin en Yvelines, France) were blotted overnight at 4°C with antibodies against β-actin (dilution 1:500; Sigma), mPGES-1 (dilution 1:200; Cayman), COX-2 (dilution 1:1,000; Cayman) or PPARγ (a gift from Professor Michel Dauça, Université Henri Poincaré, Vandœuvre-lès-Nancy, France; dilution 1:1,000), diluted in TBS-Tween with 5% bovine serum albumin. After three washings with TBS-Tween, the blot was incubated for 1 hour at room temperature with anti-rabbit IgG conjugated with horseradish peroxidase (Cell Signaling, Beverly, MA, USA) at 1:2,000 dilution in TBS-Tween containing 5% nonfat dried milk. After four washings with TBS-Tween, protein bands were detected by chemiluminescence with the Phototope Detection system in accordance with the manufacturer's instructions (Cell Signaling).

### Statistical analysis

Results are expressed as means ± SD for at least three assays. Comparisons were made by ANOVA, followed by the Fisher protected least-squares difference post-hoc test with Statview™ 5.0 software (SAS Institute Inc). A *P *value of less than 0.05 was considered significant.

## Results

### Kinetics of COX-2/mPGES-1 expression and prostaglandin production in IL-1β-stimulated rat chondrocytes

**Figure 1:**
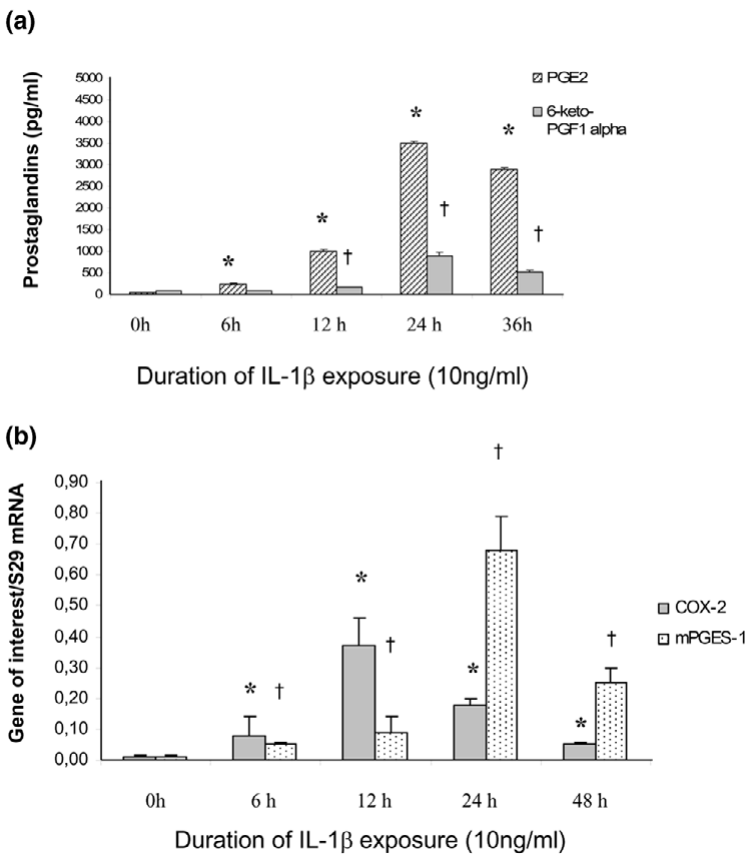
Time course of prostaglandins production, COX-2 and mPGES-1 mRNA expression, in IL-1β-stimulated chondrocytes. Rat cells were exposed to 10 ng/ml IL-1β for 6, 12, 24, 36 or 48 hours before total RNA extraction and collection of culture supernatant. **(a) **Prostaglandin levels (PGE_2_, 6-keto-PGF_1α_) assayed by ELISA in culture supernatant; **(b) **relative abundances of cyclo-oxygenase-2 (COX-2) and microsomal prostaglandin E synthase-1 (mPGES-1) mRNAs, analysed by real-time PCR and normalized to S29 mRNA. Prostaglandin levels and PCR COX-2/S29 or mPGES-1/S29 mRNA ratios presented in histograms are expressed as means ± SD for at least three independent experiments. Statistically significant differences (*P *< 0.05) from controls: * for PGE_2 _or COX-2; ^† ^for 6-keto-PGF_1α _or mPGES-1

Under basal conditions, PGE_2 _and 6-keto-PGF_1α _production was almost undetectable (Fig. 1a), whereas COX-2 and mPGES-1 mRNAs were expressed at a very low level (Fig. 1b). In response to IL-1β, PGE_2 _levels increased earlier (6 hours) than 6-keto-PGF_1α _levels (12 hours), although both peaked at 24 hours (Fig. 1a). At the time of maximal production, PGE_2 _levels were increased 70-fold and 6-keto-PGF_1α _levels 11-fold. Under these experimental conditions, COX-2 and mPGES-1 expression was induced from 6 hours, with maximal induction at 12 hours and 24 hours, respectively, after challenge with IL-1β (Fig. 1b). At these times, the extent of gene variation was higher for mPGES-1 (68-fold) than for COX-2 (37-fold).

### Effect of PPARγ agonists on prostaglandin cascade in IL-1β-stimulated rat chondrocytes

**Figure 2:**
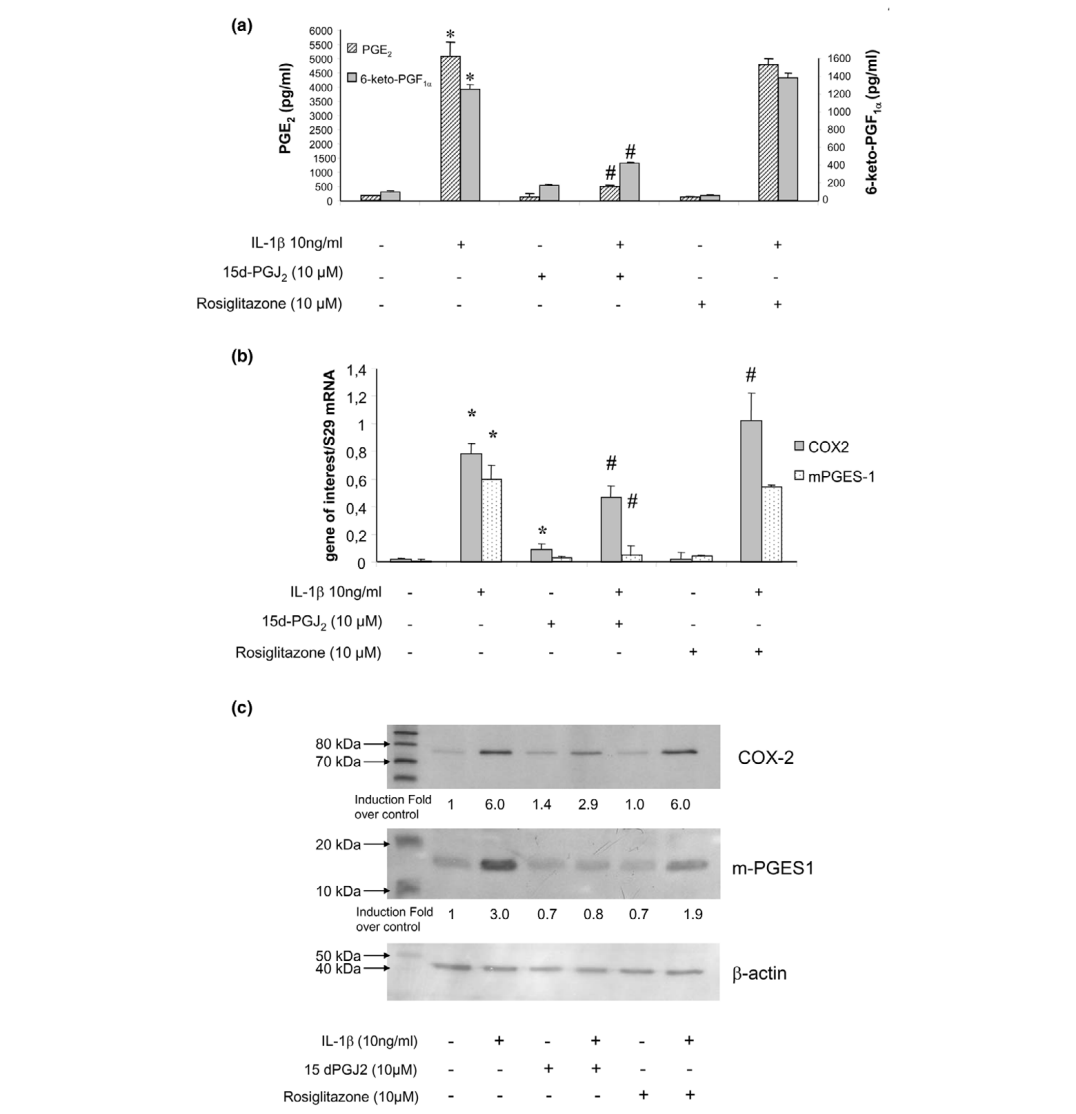
Effect of PPARγ agonists on IL-1β-induced prostaglandins levels, COX-2 and mPGES-1 mRNAs. After 4 hours of pretreatment with 10 μM 15-deoxy-Δ^12,14^prostaglandin J_2 _(15d-PGJ_2_) or rosiglitazone, chondrocytes were incubated with 10 ng/ml IL-1β for 12 or 24 hours. **(a) **PGE_2 _and 6-keto-PGF_1α _levels assayed by ELISA in culture supernatant; **(b) **relative abundances of cyclo-oxygenase-2 (COX-2) and microsomal prostaglandin E synthase-1 (mPGES-1) mRNAs, analysed by real-time PCR and normalized to S29 mRNA **(c) **COX-2 and mPGES-1 protein levels assessed by western blotting and normalized to β-actin level. Results are expressed as means ± SD for at least three independent experiments. Statistically significant differences (*P *< 0.05): *, comparison with non-stimulated controls; ^#^, comparison with IL-1β-stimulated cells.

As shown in Fig. 2a, IL-1β-induced PGE_2 _production was decreased by 92%, and 6-keto-PGF_1α _levels by 66%, by 10 μM 15d-PGJ_2_. The effect of 10 μM rosiglitazone on the stimulated levels of prostaglandins was less than the variation range of our biological system (-12% for PGE_2 _and +10% for 6-keto-PGF_1α_; Fig. 2a). Under IL-1-stimulated conditions, 10 μM 15d-PGJ_2 _decreased the expression of COX-2 and mPGES-1 by 40% and 92%, respectively, at the mRNA level (Fig. 2b) and by 52% and 73%, respectively, at the protein level (Fig. 2c). In contrast, 10 μM rosiglitazone increased COX-2 mRNAs by 37% and decreased mPGES-1 mRNAs by 10% (Fig. 2b), while leaving COX-2 protein unaffected and decreasing mPGES-1 protein by 36% (Fig. 2c). The inhibitory potency of 15d-PGJ_2 _on PGE_2 _levels was dose-related (-8% at 0.1 μM and -42% at 10 μM), whereas rosiglitazone was still ineffective at lower concentrations (-2% at 0.1 μM and -6% at 10 μM). As shown in Table 1, the proliferation of chondrocytes was increased by challenge with IL-1β but this effect was reduced neither by 15d-PGJ_2 _nor by rosiglitazone. Under IL-1-stimulated conditions, the PPARγ agonist troglitazone (10 μM) had a potency similar to that of rosiglitazone on mPGES-1 mRNAs (-12%), although its induction of COX-2 mRNAs was less (+25% versus +37%) and it was more inhibitory towards PGE_2 _levels (-25% versus -12%; data not shown). The basal levels of prostaglandins were unaffected by PPARγ agonists (Fig. 2a) despite a moderate inducing effect of 15d-PGJ_2 _on COX-2 mRNAs (Fig. 2b) and protein (Fig. 2c).

### Effect of PPARγ blockade on inhibitory potency of 15d-PGJ_2 _on stimulated prostaglandin cascade

**Figure 3:**
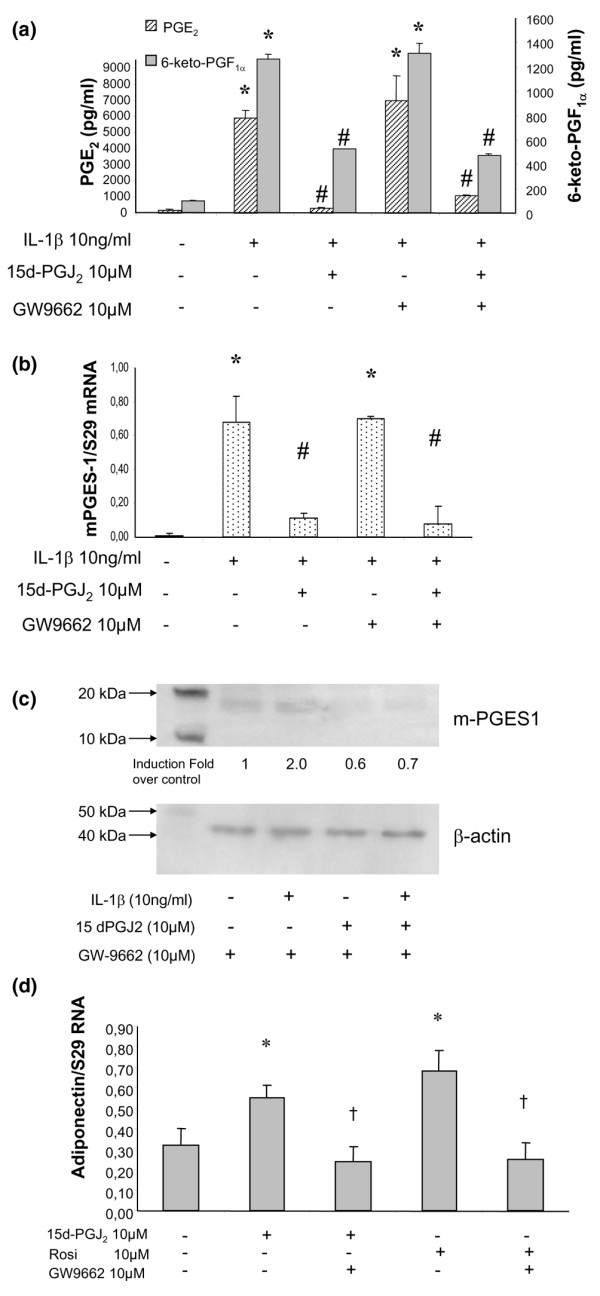
Effect of PPARγ blockade on the inhibition of IL-1β-induced responses by 15d-PGJ_2_. Chondrocytes were pretreated for 4 hours with 10 μM 15-deoxy-Δ^12,14^prostaglandin J_2 _(15d-PGJ_2_) in the presence or absence of 10 μM GW9662 (a specific antagonist of peroxisome-proliferator-activated receptor γ (PPARγ)), then stimulated with 10 ng/ml IL-1β for 24 hours before analysis of prostaglandin production and mPGES-1 expression. **(a) **PGE_2 _and 6-keto-PGF_1α _levels assayed by ELISA in culture supernatant; **(b) **relative abundance of microsomal prostaglandin E synthase-1 (mPGES-1) mRNA analysed by real-time PCR and normalized to S29 mRNA; **(c) **mPGES-1 protein level assessed by western blotting and normalized to β-actin level; **(d) **modulation of adiponectin (a PPARγ target gene) mRNAs by PPARγ ligands, analysed by real-time PCR and normalized to S29 mRNA. Results are expressed as means ± SD for at least three independent experiments. Statistically significant differences (*P *< 0.05): *, comparison with non stimulated controls; ^#^comparison with IL-1β-stimulated cells; ^†^, comparison with PPARγ agonists alone or in combination with PPARγ antagonist.

When 10 μM 15d-PGJ_2 _was tested in combination with the PPARγ antagonist GW-9662 at 10 μM, its inhibitory effect on IL-1-induced PGE_2 _(-94% versus -95%) and 6-keto-PGF_1α _(-64% versus -58%) levels remained unchanged (Fig. 3a). Similarly, the strong decrease in mPGES-1 mRNA (-93% versus -87%; Fig. 3b) and protein (-70% versus -65%; Fig. 3c) levels was unaffected. In all experiments, the inducing effect of IL-1β on prostaglandin release and gene expression was not modified by GW-9662. Because of the low efficacy of chondrocyte transfection with a PPRE-luciferase construct as a gene reporter assay, the functionality of PPARγ ligands was controlled by measuring changes in adiponectin expression. As shown in Fig. 3d, the adiponectin mRNA level was increased by 10 μM 15d-PGJ_2 _or rosiglitazone and returned to the basal level in the presence of GW-9662.

### Effect of PPARγ overexpression on inhibitory potency of 15d-PGJ_2 _on stimulated prostaglandin cascade

**Figure 4:**
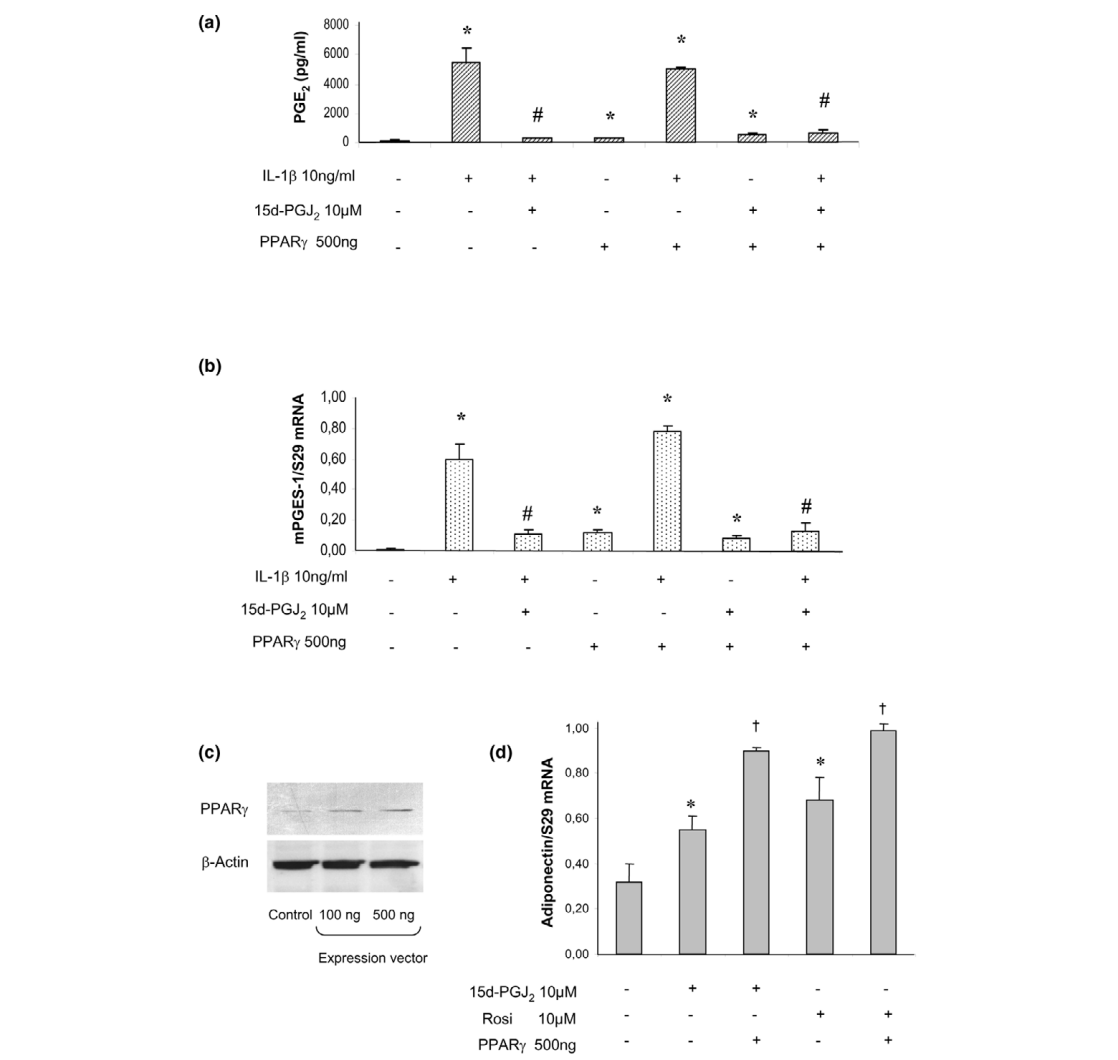
Effect of PPARγ overexpression on the inhibition of IL-1β-induced responses by 15d-PGJ_2_. Chondrocytes in six-well plates were transfected with pcDNA3.1 peroxisome-proliferator-activated receptor γ (PPARγ) construct (500 ng) for 36 hours. Thereafter, cells were pretreated for 4 hours with 10 μM 15-deoxy-Δ^12,14^prostaglandin J_2 _(15d-PGJ_2_), then stimulated with 10 ng/ml IL-1β for 24 hours before extraction of total RNA and collection of culture supernatant. **(a) **PGE_2 _levels assayed by ELISA in culture supernatant; **(b) **relative abundance of microsomal prostaglandin E synthase-1 (mPGES-1) mRNAs analysed by real-time PCR and normalized to S29 mRNA; **(c) **western blot control experiment of PPARγ and β-actin expression; **(d) **modulation of adiponectin (a PPARγ target gene) mRNAs by PPARγ agonists and pcDNA3.1 PPARγ construct, analysed by real-time PCR and normalized to S29 mRNA. Results are expressed as means ± SD for at least three independent experiments. Statistically significant differences (*P *< 0.05): *, comparison with non-stimulated controls; ^#^, comparison with IL-1β-stimulated cells; ^†^, comparison with PPARγ agonists alone or in combination with PPARγ plasmid.

Transfection of chondrocytes with a PPARγ expression vector did not change their response to IL-1β and provoked a limited increase in PGE_2 _level and mPGES-1 expression in resting cells (Fig. 4a, b). The inhibition of IL-1β-induced PGE_2 _release and mPGES-1 mRNA level by 10 μM 15d-PGJ_2 _was not impaired in cells overexpressing PPARγ (-88% versus -94% and -79% versus -82%, respectively; Fig. 4a, b). Control experiments showed that PPARγ protein was efficiently overexpressed (Fig. 4c), and that the level of adiponectin mRNA was enhanced by 15d-PGJ_2 _or rosiglitazone (Fig. 4d), in cells transfected with the PPARγ expression vector.

### Contribution of NF-κB pathway to regulation of stimulated prostaglandin cascade by IL-1β and 15d-PGJ_2 _in rat chondrocytes

**Figure 5:**
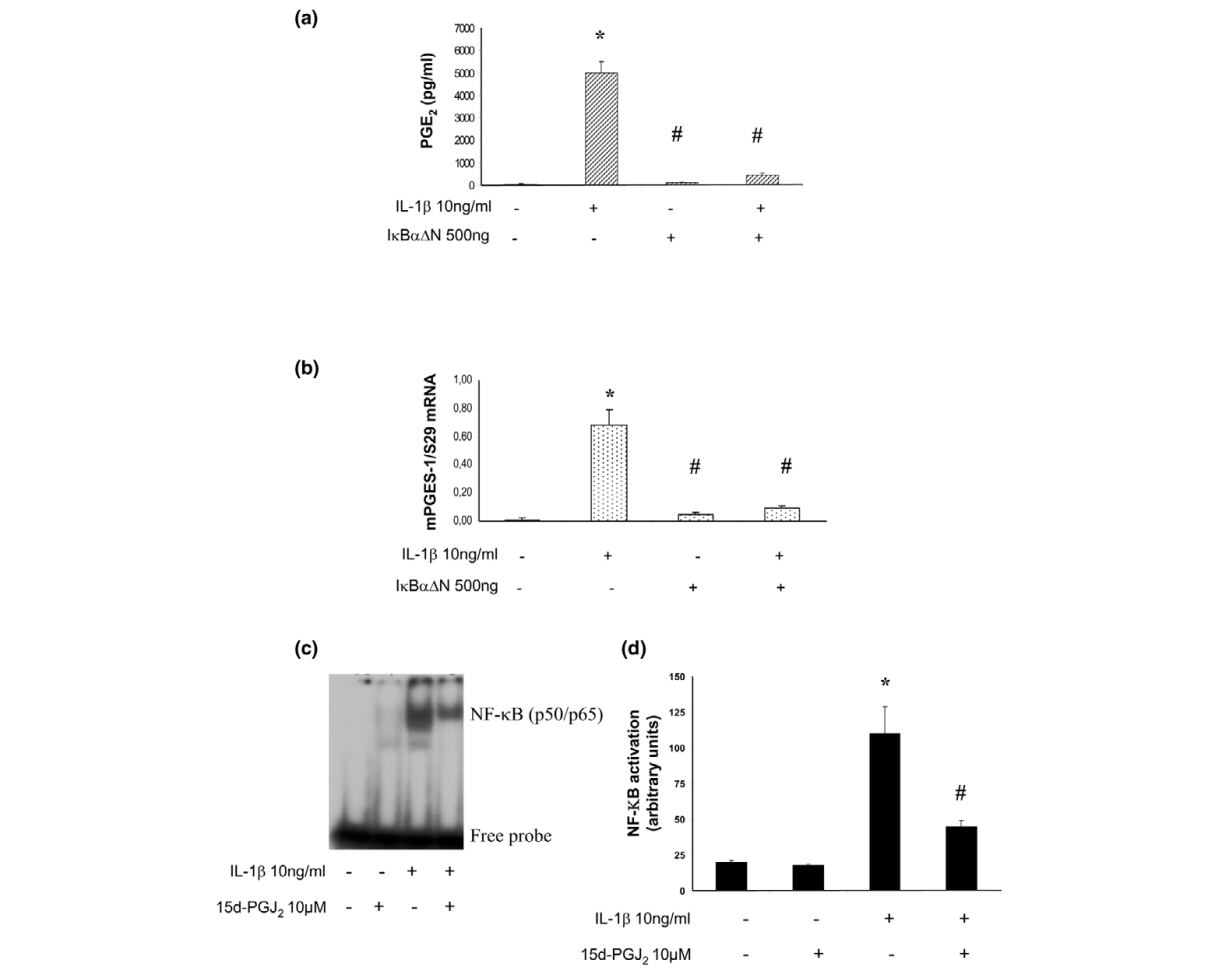
Contribution of NF-κB pathway to IL-1β-induced responses and 15d-PGJ_2 _inhibitory effects. In one set of experiments **(a, b)**, chondrocytes cultured in six-well plates were transfected with 500 ng of IKBα dominant-negative (*IκBαΔN) *vector for 24 hours, then stimulated for 24 hours with 10 ng/ml IL-1β. **(a) **PGE_2 _levels in culture supernatant assayed by ELISA; **(b) **Relative abundance of microsomal prostaglandin E synthase-1 (mPGES-1) mRNAs analysed by real-time PCR and normalized to S29 mRNA. Results are expressed as means ± SD for at least three independent experiments. In another set of experiments **(c, d)**, chondrocytes cultured in six-well plates were exposed to 10 ng/ml IL-1β for 15 min in the presence or absence of 10 μM 15-deoxy-Δ^12,14^prostaglandin J_2 _(15d-PGJ_2_) before extraction of nuclear proteins. Activation of NF-κB was determined by EMSA **(c) **and by ELISA with the TransAm^® ^technology **(d)**. Results in (d) are expressed as relative arbitrary units with IL-1β treatment set at 100, and are representative of three different experiments. Statistically significant differences (*P *< 0.05): *, comparison with non-stimulated controls; ^#^, comparison with IL-1β-stimulated cells.

As shown in Fig. 5, transfection with a dominant-negative vector of NF-κB (IκBαΔN) almost completely eliminated the synthesis of PGE_2 _(Fig. 5a) and the expression of mPGES-1 (Fig. 5b) in IL-1β-stimulated chondrocytes. As with PPARγ, transient overexpression was associated with a negligible induction of PGE_2 _and mPGES-1 in resting cells (Fig. 5a, b). Gel-shift analysis (Fig. 5c) and TransAM^® ^assay (Fig. 5d) confirmed that IL-1β induced NF-κB transactivation in rat chondrocytes and demonstrated that this activity was markedly decreased by 15d-PGJ_2_.

## Discussion

Since the discovery of a preferential coupling between several inducible enzymes of the prostaglandin cascade [31], it has become necessary to re-evaluate which step is critical for the synthesis of mediators. COX and phospholipases A_2 _have long been considered the rate-limiting enzymes; this was confirmed indirectly by the successful launching of non-steroidal anti-inflammatory drugs for the treatment of inflammation, pain and fever. However, the discovery of inducible mPGES-1 opened new insights because it was expressed at a high level in joint tissues during experimental polyarthritis [32] as well as in periarticular soft tissues and brain during acute inflammation [33]. Moreover, PGE_2 _was shown to contribute to inflammation and hyperalgesia [34], and the pivotal role of mPGES-1 in its production was confirmed by the decrease in pain nociception and inflammatory reactions in mPGES-1-deficient mice [35]. Finally, in contrast with COX inhibition, blockade of mPGES-1 could theoretically favour the biotransformation of cyclic endoperoxide H_2 _into anti-inflammatory 15d-PGJ_2 _depending on the tissue expression of PGD synthase [36]. The pathophysiological role of mPGES-1 in inflammatory diseases is therefore worthy of study, and inhibitors of this enzyme might have potent therapeutical relevance [37].

In the present study we investigated first the respective time courses of prostaglandin production and induction of genes of the arachidonic acid cascade in chondrocytes activated with IL-1β, a pro-inflammatory cytokine with a central function in joint diseases [38]. We confirmed that normal rat chondrocytes were very sensitive to stimulation by IL-1β and produced large amounts of prostaglandins [39], with kinetics comparable to that of human osteoarthritic chondrocytes [19,40] or the immortalized T/C-28a2 cell line [41]. As expected, resting and activated chondrocytes produced several types of prostaglandin, although the extent of variation was much higher for PGE_2 _than for 6-keto-PGF_1α _[39,42]. Although IL-1β-induced PGE_2 _synthesis was associated with the induction of COX-2 expression in articular cells [19,39], it has been shown that COX-2 and mPGES-1 are coordinately upregulated, but with different time courses [37,39,43], and that their subcellular localizations overlap in the perinuclear region [40,43]. Our kinetics study confirmed an early induction of COX-2 and a delayed induction of mPGES-1 in IL-1β-stimulated chondrocytes [40], thereby mimicking the time course reported for inflamed rat tissues [33].

The increase in PGE_2 _level fitted well with the extent of mPGES-1 gene induction but not with that of COX-2, whereas changes in the 6-keto-PGF_1α _level were much smaller than the extent of COX-2 induction. Of course, each inducible enzyme of the arachidonic acid cascade is rate limiting in that it controls the bioavailability of substrate to downstream effectors [6,7]. However, our results strongly support the contention that mPGES-1 expression is the most limiting step in PGE_2 _synthesis, consistent with previous experiments with MK-886 [40], a five-lipoxygenase activating protein (FLAP) inhibitor with *in vitro *inhibitory potency towards mPGES-1 [44].

Because the stimulated synthesis of 6-keto-PGF_1α _requires successive metabolization by COX-2 and prostacyclin synthase (PGIS), the lower than expected increase could reflect a limited induction of PGIS in rat chondrocytes. Thus, induction of PGIS by IL-1β was less than double that in rat non-articular cells [45] despite its selective upregulation by COX-2 induction in human endothelial cells [46]. A decrease in PGIS expression, contrasting with an increase in mPGES-1 expression, was also reported in inflamed tissues of rat with adjuvant polyarthritis [32]. Alternatively, other metabolic pathways might have been favoured such as the conversion of cyclic endoperoxides into other prostaglandins [42], depending on the substrate concentration dependences of the terminal synthases [6,46]. Arachidonic acid could also have been transformed into hydroxylated non-prostaglandin metabolites, which can be synthesized in IL-1β-stimulated chondrocytes [25], depending on the balance between the COX and lipoxygenase pathways [47]. In all instances, IL-1β stimulated all inducible steps of the arachidonic acid cascade to produce PGE_2 _maximally in rat chondrocytes.

The study of the expression of COX-2 or mPGES-1 and the release of prostaglandins in activated chondrocytes showed that 15d-PGJ_2 _was strongly inhibitory, whereas the high-affinity PPARγ agonist rosiglitazone was marginally potent in the same concentration range. Although 15d-PGJ_2 _and rosiglitazone were able to induce adiponectin expression, thereby demonstrating their potency to activate PPARγ, these results, irrespective of the binding affinity of agonists to PPARγ [48], supported the idea that this isotype was not primarily involved. It is interesting to note that the inducing effect of rosiglitazone on COX-2 mRNA was not confirmed at the protein level and that it was slightly inhibitory on mPGES-1, resulting in an unchanged PGE_2 _level. When we tried to decrease the inhibitory potency of 15d-PGJ_2 _by antagonizing its binding to PPARγ with GW-9662, we failed to observe any changes in gene mRNAs and PGE_2 _levels. As a corollary, the efficient overexpression of PPARγ did not enhance the potency of 15d-PGJ_2 _in our experimental system. Finally, despite the existence of a PPRE consensus site in the promoter of human COX-2 [49] and evidence that 15d-PGJ_2 _stimulates COX-2 gene expression in rat chondrocytes as in human synovial fibroblasts [50], we failed to observe any change in the basal production of PGE_2_, as reported previously in human osteoarthritic chondrocytes [51].

Taken together, our data strongly support the contention that 15d-PGJ_2 _was acting independently of PPARγ. Very few data are available in the rat species, but a PPARγ-dependent inhibition of inducible arachidonic acid cascade was reported in cardiac myocytes stimulated with IL-1β [52]. Because the inhibitory potency of 15d-PGJ_2 _on the COX-2, mPGES-1 and PGE_2 _levels was closely similar in both studies, we suggest that this discrepancy might be supported by cell type specificities. Indeed, the decrease in the levels of prostacyclin metabolites was different between cardiac myocytes and chondrocytes (no inhibition versus -66%) for a comparable extent of COX-2 inhibition (-40 to -50%), whereas the synthetic PPARγ agonist troglitazone was much more inhibitory towards PGE_2 _levels in the former cell type. In human chondrocytes, the inhibitory potency of 15d-PGJ_2 _was similar to our results on PGE_2 _levels [51], although supported by a stronger inhibition of COX-2 and a PPARγ-dependent inhibition of mPGES-1 [53]. In this cell type, the dose-dependent effect of 15d-PGJ_2 _was also thought to be mainly supported by the activation of PPARγ for the control of other inflammatory mediators [54] and apoptosis [55]. The biological responses to PPAR agonists are well known to differ between species [56], but our data support the notion that the potency of PPARγ agonists on joint cells might be influenced by differences in both cell type and species. Consistently, 15d-PGJ_2 _and troglitazone were shown to inhibit PGE_2 _production and mPGES-1 expression in IL-1β-stimulated human synovial fibroblasts [57], whereas troglitazone was totally ineffective on LPS-induced COX-2 expression in rat cells [20]. Finally, one could underline that the contribution of PPARγ might also depend on 15d-PGJ_2 _concentration, because the inhibition of PGE_2 _production was reported to be PPARγ-dependent in the nanomolar range while becoming PPARγ-independent in the micromolar range [58]. Despite a variable contribution of the PPARγ isotype depending on the biological system used, the present study confirms that 15d-PGJ_2 _downregulates inducible steps of the arachidonic acid cascade in joint cells, thereby probably contributing to its anti-arthritic properties [10].

The inhibitory potency of 15d-PGJ_2 _was PPARγ-independent but dose-related, which does not favour non-specific activity. This led us to investigate whether 15d-PGJ_2 _could interact with the NF-κB pathway, which is known to be one of its major targets in many cell types [59,60]. A previous study of the mouse mPGES-1 promoter indicated that it lacked binding sites for NF-κB, the cAMP-response element, and E-box, which have been implicated in COX-2 induction, implying that the mechanisms for inducible expression of COX-2 and mPGES-1 were distinct in this species [61]. In human synovial fibroblasts, transcriptional regulation of the mPGES-1 gene by IL-1β was shown to be closely dependent on the transcription factor early growth response factor-1 (Egr-1) [57], although activator protein-1 and specificity protein-1 binding sites were also found [62]. In human chondrocytes, IL-1β was demonstrated to use overlapping, but distinct, signalling pathways to induce COX-2 and mPGES-1, with a major role for ERK1/2 and p38β MAPK in controlling the latter [41]. However, in a non-articular human cell type, a substantial role for NF-κB was demonstrated recently in the coordinate induction of COX-2 and mPGES-1 by IL-1β [63]. As indicated previously, some of these signalling pathways can be inhibited in a PPARγ-dependent manner, possibly secondary to the squelching of transcription cofactors such as CBP/p300 by protein-protein interaction with PPARγ [64]. Consequently, such a mechanism is unlikely to explain the PPARγ-independent inhibitory potency of 15d-PGJ_2 _in our system.

Although the promoter of rat mPGES-1 has not so far been explored, our data with mutated IκBα are consistent with a major role of NF-κB in the control of its transcriptional activity. We showed further that 15d-PGJ_2 _inhibited IL-1β-induced NF-κB nuclear binding (with the use of EMSA) and transactivation (with a TransAM^® ^assay). This inhibitory effect was consistent with the ability of 15d-PGJ_2 _to decrease IκB kinase (IKK) activity, by limiting the phosphorylation of its catalytic subunit IKKβ, and to prevent IκBα degradation by the proteasome [65]. Because of the high chemical reactivity of its cyclopentenone ring with substances containing nucleophilic groups, such as the cysteinyl thiol group of proteins [66], possible mechanisms may include covalent binding of 15d-PGJ_2 _to IKK [67] or alkylation of a conserved cysteine residue located in the p65 subunit DNA-binding domain of NF-κB [68]. A possible chemical interaction with NF-κB components is further sustained by the ability of 15d-PGJ_2 _to suppress the induction of COX-2 in PPARγ-deficient macrophages [14]. However, we did not investigate whether NF-κB binds directly to mPGES-1 rat promoter, and the delayed induction of mPGES-1 by IL-1β supports indirect regulation. NF-κB was consistently shown to regulate the early expression of Egr-1 [69], which has been implicated in the regulation of murine and human mPGES-1 [57,61]. Alternatively, we cannot exclude the possibility that inhibition of COX-2 by 15d-PGJ_2 _might participate partly in its inhibitory potency towards mPGES-1, because PGE_2 _production associated with COX-2 is involved in the induction of mPGES-1 by IL-1β in rheumatoid synovial fibroblasts [43].

## Conclusion

The data reported here demonstrate that IL-1β activates COX-2 and mPGES-1 sequentially in rat chondrocytes and that the production of large amounts of PGE_2 _depends mainly on the expression of mPGES-1. In our cell type, 15d-PGJ_2 _displayed a strong inhibitory effect on prostaglandin levels and gene expression, whereas rosiglitazone was poorly active in the same concentration range. Despite its efficient activation of PPARγ, the effect of 15d-PGJ_2 _occurred through a PPARγ-independent mechanism. The activation of the NF-κB pathway was critical for mediating the inducing effect of IL-1β on PGE_2 _levels and mPGES-1 expression in rat chondrocytes, and was abolished by 15d-PGJ_2_. On the basis of the pathophysiological role of PGE_2 _in rheumatic diseases, our data support the general meaning that 15d-PGJ_2 _could behave as an endogenous regulator of inflammation if it was synthesized in sufficient amounts within joint tissues.

## Abbreviations

15d-PGJ_2 _= 15-deoxy-Δ^12,14^prostaglandin J_2_; COX-2 = cyclo-oxygenase-2; DMEM = Dulbecco's modified Eagle's medium; DTT = dithiothreitol; EMSA = electrophoretic mobility-shift assay; FCS = fetal calf serum; IKK = IκB kinase; IL = interleukin; mPGES-1 = microsomal prostaglandin E synthase-1; NF-κB = nuclear factor-κB; PBS = phosphate-buffered saline; PCR = polymerase chain reaction; PG = prostaglandin; PGI_2_, prostacyclin; PGIS = prostacyclin synthase; PPAR = peroxisome-proliferator-activated receptor; PPRE = peroxisome-proliferator-response element.

## Competing interests

The author(s) declare that they have no competing interests.

## Authors' contributions

AB and DM performed the molecular studies and drafted the manuscript. SS and MK performed the immunoassays and the statistical analysis. MMG and PN supervised the study design and the manuscript. BT and JYJ conceived the study and participated in its design and final presentation. All authors read and approved the final manuscript.

## Figures and Tables

**Table 1:** Effects of peroxisome-proliferator-activated receptor γ agonists on viability of IL-1β-stimulated chondrocytes

Agonist added	*A*_580_	Percentage of control
None (control)	0.81 ± 0.05	100
IL-1β (10 ng/ml)	1.12 ± 0.04*	138
15d-PGJ_2 _(10 μM)	0.91 ± 0.07	112
IL-1β + 15d-PGJ_2_	1.10 ± 0.05	135
Rosiglitazone (10 μM)	0.95 ± 0.09	117
IL-1β + rosiglitazone	1.12 ± 0.07	138

15d-PGJ_2_, 15-deoxy-Δ^12,14^prostaglandin J_2_.

*, *P *< 0.05, comparison with non-stimulated controls
